# The developmental regulator PKL is required to maintain correct DNA methylation patterns at RNA-directed DNA methylation loci

**DOI:** 10.1186/s13059-017-1226-y

**Published:** 2017-05-31

**Authors:** Rong Yang, Zhimin Zheng, Qing Chen, Lan Yang, Huan Huang, Daisuke Miki, Wenwu Wu, Liang Zeng, Jun Liu, Jin-Xing Zhou, Joe Ogas, Jian-Kang Zhu, Xin-Jian He, Heng Zhang

**Affiliations:** 1 Shanghai Center for Plant Stress Biology, Shanghai Institutes for Biological Sciences, Chinese Academy of Sciences, Shanghai, 210602 China; 2 National Institute of Biological Sciences, Beijing, 102206 China; 3 Department of Biochemistry, Purdue University, West Lafayette, IN 47907 USA; 4 Department of Horticulture and Landscape Architecture, Purdue University, West Lafayette, IN 47907 USA

**Keywords:** Non-coding RNA (ncRNA), ATP-dependent chromatin remodeling, DNA methylation, RNA-directed DNA methylation (RdDM)

## Abstract

**Background:**

The chromodomain helicase DNA-binding family of ATP-dependent chromatin remodeling factors play essential roles during eukaryote growth and development. They are recruited by specific transcription factors and regulate the expression of developmentally important genes. Here, we describe an unexpected role in non-coding RNA-directed DNA methylation in *Arabidopsis thaliana*.

**Results:**

Through forward genetic screens we identified *PKL*, a gene required for developmental regulation in plants, as a factor promoting transcriptional silencing at the transgenic *RD29A* promoter. Mutation of *PKL* results in DNA methylation changes at more than half of the loci that are targeted by RNA-directed DNA methylation (RdDM). A small number of transposable elements and genes had reduced DNA methylation correlated with derepression in the *pkl* mutant, though for the majority, decreases in DNA methylation are not sufficient to cause release of silencing. The changes in DNA methylation in the *pkl* mutant are positively correlated with changes in 24-nt siRNA levels. In addition, *PKL* is required for the accumulation of Pol V-dependent transcripts and for the positioning of Pol V-stabilized nucleosomes at several tested loci, indicating that RNA polymerase V-related functions are impaired in the *pkl* mutant.

**Conclusions:**

*PKL* is required for transcriptional silencing and has significant effects on RdDM in plants. The changes in DNA methylation in the *pkl* mutant are correlated with changes in the non-coding RNAs produced by Pol IV and Pol V. We propose that at RdDM target regions, *PKL* may be required to create a chromatin environment that influences non-coding RNA production, DNA methylation, and transcriptional silencing.

**Electronic supplementary material:**

The online version of this article (doi:10.1186/s13059-017-1226-y) contains supplementary material, which is available to authorized users.

## Background

DNA methylation is an important epigenetic modification that is associated with heterochromatin formation and transcriptional gene silencing. Plant DNA methylation occurs in three different sequence contexts: CG, CHG, and CHH (H = A, C, T). DNA methylation patterns are faithfully replicated from generations to generations [1]. Maintenance of CG methylation requires the DNA methyltransferase MET1 [2, 3] and the VIM/UHRF1 proteins [4, 5], which function at the DNA replication foci to copy methylation from the parent strand to the daughter strand. Maintenance of CHG methylation requires the DNA methyltransferase CMT3 [6] and the histone methyltransferase KYP/SUVH4, SUVH5, and SUVH6 [7], through a positive feedback loop that involves H3K9me2 [8, 9]. The RNA-directed DNA methylation (RdDM) pathway (recently reviewed in [10, 11]) and another DNA methyltransferase CMT2 [12, 13] are required to maintain CHH methylation.

RdDM is also required for de novo methylation in all three sequence contexts [6]. Genetic screens and biochemical approaches have identified more than 40 proteins involved in RdDM thus far [10]. RdDM requires two classes of non-coding RNAs: the 24-nucleotide (24-nt) small interfering RNAs (siRNA) whose production is initiated by RNA polymerase IV (Pol IV) and the scaffold RNAs that is generated by RNA polymerase V (Pol V). Both Pol IV and Pol V evolved from RNA polymerase II and the three share six common subunits out of 12 [11, 14, 15]. Loading of siRNAs into the Argonaute (AGO4/6) proteins and base pairing between the siRNAs and scaffold RNAs are believed to provide the target information for de novo methylation by DRM2 [16].

RdDM is involved in many biological processes, including repression of transposon activity, response to biotic and abiotic stresses, paramutation, establishment of methylation patterns during reproduction (recently reviewed in [10]). Despite its important functions in de novo methylation, most Arabidopsis *RdDM* mutants do not have obvious developmental phenotypes. Indeed, only *rdm4/dms4* exhibits developmental defects among all the RdDM mutants reported in Arabidopsis [17, 18]. In addition to being a transcriptional regulator of Pol IV and Pol V, RDM4/DMS4 is also involved in Pol II function, thus influencing the expression of developmentally important genes [18].

ATP-dependent chromatin remodeling factors belong to the SF2 superfamily of DNA helicases [19]. As the name suggests, they utilize energy from ATP hydrolysis to modify the conformation of nucleosomes and chromatin. In vivo they usually exist in the form of multi-subunit protein complexes [20]. A comprehensive phylogenetic analysis using the ATPase domain sequences identified seven large groups and 24 subfamilies in all eukaryotes [19]. The Arabidopsis genome contains members in 18 out of these 24 subfamilies. At least four subfamilies have been suggested to function in DNA methylation regulation but their molecular mechanisms remain to be clarified. DDM1, the first remodeling factor that was identified to affect DNA methylation in plants, plays a major role in promoting transposon methylation. Loss of *DDM1* leads to elimination of almost all DNA methylation in heterochromatic regions [21]. DDM1 facilitates DNA methylation by assisting DNA methyltransferases to access the most repressed chromatin [12]. The plant-specific DRD1 subfamily is specialized for the RdDM pathway. The six-member subfamily contains four CLSY proteins and two DRD proteins. DRD1 is part of a three-component complex called DDR (DRD1/DMS3/RDM1) that assists in RNA Pol V transcription [22–24]. The function of its closest homolog DRD2 remains unclear. CLSY1 is required for siRNA accumulation and is believed to have a role in assisting Pol IV transcription [25]. The interactions between Pol IV and CLSY and between Pol V and DRD were detected in both Arabidopsis and maize [23, 26–28]. In addition, a SWI/SNF complex that belongs to the Snf2 subfamily functions downstream of Pol V-generated scaffold RNAs through its interaction with the IDN complex and promotes methylation of RdDM targets [29]. Recently the ETL1/CHR19 remodeler and two proteins of the five-member Ris1 subfamily, FRG1/CHR27 and FRG2/CHR28, were also found to be required for DNA methylation and silencing at some RdDM loci [30, 31].

PKL belongs to the Mi-2/CHD3 subfamily of ATP-dependent chromatin remodelers [19, 32]. PKL was originally identified as a factor that is required to repress embryonic traits during seed germination and to facilitate the transition from embryonic phase to vegetative phase of plants [33]. Later it was identified in multiple suppressor screens and was found to be involved in establishment of carpel polarity, initiation of lateral roots, and promoting hypocotyl cell elongation during skotomorphogenesis [34–36]. The *pkl* mutant exhibits pleiotropic defects including semi-dwarfism, reduced apical dominance, decreased root meristem activity, and other developmental phenotypes [33, 37]. PKL may also play a role in integrating hormone signaling during plant development [33, 38]. PKL mainly exists as a monomer in plant cells, and it exhibits in vitro nucleosome remodeling activity [32]. In contrast, its animal homolog Mi-2 forms stable complexes with histone deacetylases (HDAC) called NURD, which account for the highest HDAC activity in human cells [39–42]. Although Mi-2/CHD3 proteins mainly function as a transcriptional co-repressor, instances of these proteins being recruited by specific transcription factors and functioning as transcription co-activator were also reported in specific cell types [43, 44]. Similarly, PKL functions as a transcriptional repressor in many cases and is required to promote H3K27me3, a repressive histone modification typically associated with tissue-specific genes [45, 46], but it was also found that PKL could promote the transcription of specific genes by interacting with transcriptional activators [36]. Overall the CHD3-type chromatin remodeling factors are employed as transcriptional co-regulators in many important developmental processes [47].

Besides developmental genes, PKL was also found to bind directly to certain transposable elements [45], although microarray-based transcriptome analyses did not identify significant overlaps with other DNA methylation mutants [46]. Thus, whether and how PKL functions in heterochromatic regions remain largely unknown. In this study, we identified a role of PKL in RNA-directed DNA methylation. In genetic screens searching for mutants defective in transcriptional silencing of the *pRD29A-LUC* transgene, we identified two alleles of *rdm18*, both of which showed defects in DNA methylation and silencing of a subset of classical RdDM target loci. Map-based cloning revealed that the *rdm18* mutations reside in the *PKL* gene. Based on whole genome DNA methylation, small RNA, and transcriptome analyses, we propose that PKL may create a chromatin environment that influences non-coding RNA transcription, DNA methylation, and transcriptional silencing through its nucleosome remodeling activity. These results reflect the complexity in transcriptional regulation of non-coding RNAs and show that the developmentally important chromatin remodeler PKL also plays a role in RNA-directed DNA methylation.

## Results

### RDM18 is required for silencing of the pRD29A-LUC transgene

**Fig. 1:**
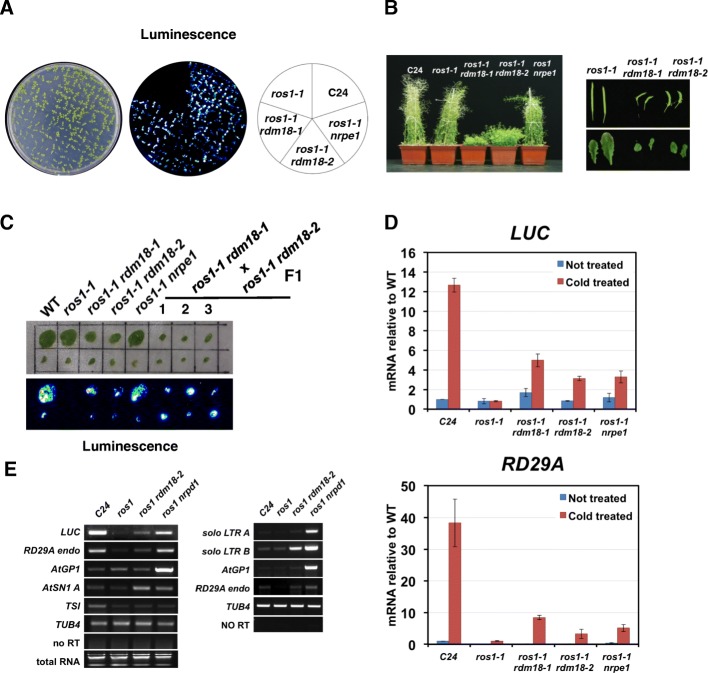
*RDM18* promotes transcriptional gene silencing at RdDM loci. **a** Bioluminescence phenotype of two-week-old *ros1 rdm18* seedlings. **b** The *ros1 rdm18* mutants exhibit multiple developmental defects. Shown in the figure includes dwarfism, short and curled siliques, and small leaves (eight-week-old plants). **c** Bioluminescence phenotype of F1 plants generated from crosses between *ros1-1 rdm18-1* and *ros1-1 rdm18-2*. Cauline leaves from six-week-old plants were used for the analyses. **d** Transcript levels of the *pRD29A-LUC* transgene and endogenous *RD29A* gene examined by quantitative reverse transcription polymerase chain reaction (qRT-PCR). Relative transcript levels were shown with non-treated C24 set to one. *Error bars* indicate standard deviations calculated from three biological replicates. **e** Transcript levels of typical RdDM loci measured by RT-PCR. Two independent RT-PCR experiments were performed and the results are shown in two separate panels. *LUC:* transgene *pRD29A-LUC*, *RD29A endo:* endogenous *RD29A* gene. Ethidium bromide stained agarose gel (total RNA) and no reverse transcriptase PCR (no RT) serve as the loading control and the negative control respectively

The *RD29A* promoter is abiotic stress responsive and is activated when the plant is under cold or salinity. We previously showed that the transcriptional activity of a *pRD29A-LUC* transgene is regulated by DNA methylation [48]. The 5-methylcytosine DNA glycosylase ROS1 is required to prevent DNA methylation at the *RD29A* promoter and allows gene activation [49]. By screening for mutants that regain luminescence signals in the *ros1-1* mutant background, a number of factors that are involved in RNA-directed DNA methylation (RdDM) were identified [50]. From a T-DNA mutagenized pool of *ros1-1*, we identified a mutant named *rdm18-1* that exhibited strong luminescence signals after cold treatment (Fig. 1a). The *rdm18-1* mutant also exhibited developmental defects including dwarfism, late flowering, small and curled leaves, and severely reduced fertility (Fig. 1b). In a separate ethyl methanesulfonate (EMS) mutagenized pool, we identified a second *rdm18* mutant allele (*rdm18-2*) that exhibited increased luminescence signals, as well as similar developmental defects as *rdm18-1* (Fig. 1a and b). The intensity of the luciferase signal in *ros1 rdm18* double mutants is comparable to that of *ros1 nrpe1*, which serves as a positive control (Fig. 1a). In order to determine whether *rdm18-1* and *rdm18-2* are allelic, we made crosses between the two mutants. The F1 plants also exhibited increased luminescence signals and various developmental defects as the parents, indicating that the two mutations reside in the same gene (Fig. 1c).

We observed changes at the transcript level for both the transgenic *pRD29A-LUC* and endogenous *RD29A* genes. Quantitative reverse transcription polymerase chain reaction (qRT-PCR) identified substantially higher levels of *LUC* transcripts in *ros1-1 rdm18* mutants compared to *ros1-1* (Fig. 1d). The EMS induced *rdm18-2* mutant seems to be a weaker allele because its luciferase signals are weaker compared to *ros1-1 rdm18-1* (Fig. 1a) and the adult plants are slightly taller than *ros1-1 rdm18-1* (Fig. 1b). We also observed less *LUC* transcripts in *ros1-1 rdm18-2* than in *ros1-1 rdm18-1* (Fig. 1d). Similar to previously identified RdDM mutants, the endogenous *RD29A* gene also showed released silencing in the *ros1-1 rdm18* double mutants compared to *ros1-1* (Fig. 1d).

In addition to the *pRD29A-LUC* transgene, the same T-DNA insertion also contains a *p35S-NPTII* (neomycin phosphotransferase) transgene, which is expressed in wild-type (WT) C24 plants and confers kanamycin resistance. When the *ROS1* gene is mutated, the *35S* promoter gained more DNA methylation and became silenced [49]. We examined whether *RDM18* could also play a role in the silencing of the *NPTII* gene. The *ros1-1* plants are sensitive to kanamycin, while the *rdm18-1 ros1-1* mutant is partially resistant (Additional file 1: Figure S1A). Due to the severe developmental defects, the *ros1-1 rdm18* seedlings are generally much smaller on the plate (Additional file 1: Figure S1A). Consistent with the partial gain of kanamycin resistance, we observed elevated levels of *NPTII* transcript in the *ros1-1 rdm18-1* double mutant compared to *ros1-1* (Additional file 1: Figure S1B). This is different from classical RdDM components such as *NRPD1* and *NRPE1*, which are not required for the silencing of the *NPTII* gene in the *ros1* background [51].

We next examined other genomic loci that are also regulated by RdDM. Using RT-PCR, we detected increased levels of transcripts at *AtSN1* and *soloLTR B* in the *ros1 rdm18* double mutants compared to *ros1* (Fig. 1e). However, no changes were observed for *AtGP1* or *TSI* (Fig. 1e).

In summary, we identified two *rdm18* alleles that showed defects in silencing of the *pRD29A-LUC* transgene and some endogenous RdDM targets. Different from previously identified RdDM mutants, *RDM18* is required for multiple developmental processes and also plays a role in promoting silencing of the *p35S-NPTII* transgene.

### RDM18 is required for DNA methylation at selected RdDM targets

In order to test the involvement of *RDM18* in DNA methylation regulation, we measured DNA methylation levels of both transgenic and endogenous RdDM targets using multiple methods. Sodium bisulfite sequencing revealed mild decreases in non-CG methylation levels at the transgenic *RD29A* promoter in *ros1-1 rdm18* double mutants compared to *ros1-1* (Additional file 1: Figure S2A). However no consistent changes at the endogenous *RD29A* promoter were observed (Additional file 1: Figure S2A). Decreases in non-CG methylation were detected at the *AtSN1* transposon in the *ros1-1 rdm18* mutant (Additional file 1: Figure S2B), correlated with released silencing of this locus (Fig. 1e). However, at two other known RdDM loci, *AtMu1* (a MULE transposon) and *MEA-ISR* (*MEDEA INTERSTITIAL SUBTELOMERIC REPEATS*), no changes in DNA methylation levels were found (Additional file 1: Figure S2C).

We used southern blotting to examine DNA methylation levels at the 5S ribosomal DNA repeats and centromeric regions. The *ros1-1 rdm18* mutant showed slightly reduced DNA methylation at the 5S rDNA repeats, albeit not to the same level as in *ros1-1 nrpd1* (Additional file 1: Figure S2D). Mutation of *RDM18* had no effect on the methylation levels of 180-bp centromeric repeats, similar to the *ros1-1 nrpd1* control (Additional file 1: Figure S2E).

The expression level of the demethylase gene *ROS1* is significantly decreased in plants that are defective in DNA methylation [52–54]. A methylation monitoring sequence (MEMS) was found within the promoter region of *ROS1*, methylation of which correlated with increased *ROS1* expression [54, 55]. Thus, the transcript level of *ROS1* may serve as an indicator of the DNA methylation activity in the cell. We observed a threefold to fourfold decrease of *ROS1* transcripts in *ros1-1 rdm18* mutants, similar to that in *ros1-1 nrpe1* (Additional file 1: Figure S2F). Overall these results indicate that *RDM18* is required for proper DNA methylation at some RdDM loci.

### Map-based cloning of RDM18

**Fig. 2:**
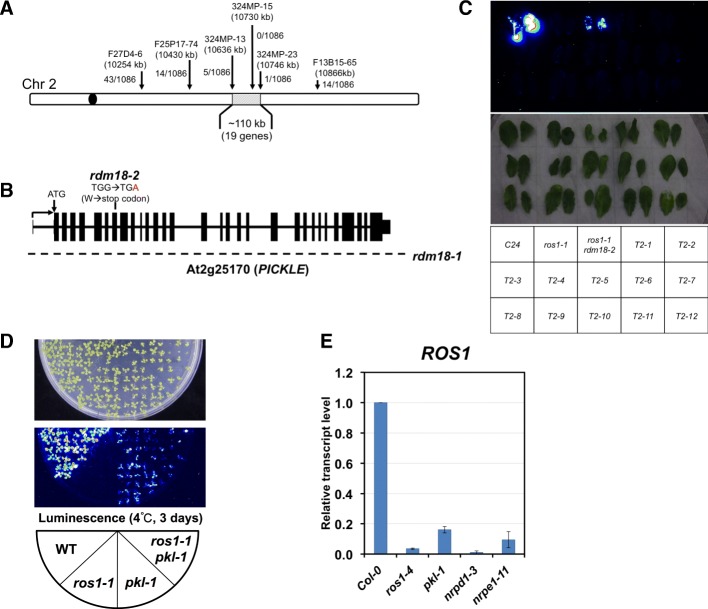
Map-based cloning of the *rdm18* mutations. **a** A *diagram* showing the mapped genomic region of *rdm18-1*. Genetic markers and their positioning on the chromosome are indicated on top of the *arrow*. **b** A *diagram* showing the gene structure of *PKL* and mutations identified in the *rdm18* mutants. The *dashed line* indicates a whole gene deletion identified in the *rdm18-1* mutant. **c** Bioluminescence phenotype of the T2 plants from the PKL-FLAG transformation of *ros1-1* (–/–) *rdm18-2* (+/–) plants. **d** The *pkl-1* mutation released silencing at the *RD29A* promoter in the *ros1-1* background. The F3 seedlings with indicated genotypes from *pkl-1* (Col) x *ros1-1* (C24) crosses were subjected to luminescence imaging after cold treatment for three days. **e** The transcript level of the *ROS1 gene* decreases in the *pkl-1* mutant. Relative transcript level measured by real-time PCR is shown and the level in WT (Col-0) is arbitrarily set to 1. *Error bars* represent standard deviations calculated from three biological replicates

We used map-based cloning to identify the causal mutation in both *rdm18* alleles. We narrowed the *rdm18-1* mutation down to a ~110-kb region on chromosome 2 (Fig. 2a). Screening of genes with decreased expression in that region identified *PICKLE* (*PKL*, At2g25170), a chromatin remodeling factor gene involved in multiple developmental processes. Consistent with the observation that *rdm18* mutants exhibit severe developmental defects, pleiotropic developmental phenotypes of the *pkl* mutant was reported [33, 34]. However, the *pkl-1* mutant, which was a strong loss-of-function mutant allele from the *Col* ecotype, was taller and produced more seeds, indicating that different genetic backgrounds of *C24* and *Col* may contribute to the difference.

T-DNA insertion in the *rdm18-1* mutant caused a deletion that spans at least the whole PKL gene body, as using 15 primer pairs that tiled the gene body failed to generate any PCR products (Fig. 2b; data not shown). The mutation of *rdm18-2* is a G-A point mutation in the eighth exon of the PKL gene, which changes a tryptophan residue (W342) to a premature stop codon in the protein sequence (Fig. 2b).

In order to further confirm that the *rdm18* mutations reside in *PKL*, we transformed the *rdm18* mutants with constructs that contain the *PKL* genomic DNA fragment. Due to severe fertility phenotype of *rdm18* mutants, we failed to generate any complementation lines despite multiple attempts. Thus, we transformed the *PKL-FLAG* genomic constructs [45] into *ros1-1 +/+ rdm18-2 +/–* plants, which were generated by crossing *ros1-1 rdm18-2* to *ros1-1*. The *rdm18-2* allele was used because the point mutation allowed us to distinguish homozygous from heterozygous alleles. After transformation, we obtained two T1 plants that were heterozygous for the *rdm18-2* mutation. In the following T2 generation, we selected glufosinate-resistant plants for genotyping. Though genotyping confirmed that the presence of *PKL-FLAG* transgene and that the *rdm18-2* mutation segregated (data not shown), all the plants exhibited no luciferase signals or developmental defects (Fig. 2c), indicating the presence of the *PKL-FLAG* transgene complemented the mutant phenotype.

We also crossed *pkl-1* (in the Col background) to *ros1-1* (in the C24 background) to confirm that *pkl-1 ros1-1* could recapitulate the mutant phenotype of *ros1-1 rdm18*. We examined the phenotype in F3 progenies of the cross. We found that in homozygous *pkl-1* or *pkl-1 ros1-1* plants containing the *pRD29A-LUC* transgene emitted luminescence signals whereas *ros1-1* plants did not (Fig. 2d), indicating that the *pkl-1* mutation could suppress the silencing of *pRD29A-LUC* in the *ros1-1* mutant background.

We also examined the *ROS1* transcript level in the *pkl-1* mutant using qRT-PCR. Similar to those in *nrpe1* and *rdm18* mutants (Additional file 1: Figure S2F), *ROS1* transcripts decreased to less than 20% of WT level in the *pkl-1* mutant (Fig. 2e). These results indicate that the mutation of *PKL* is responsible for the silencing defects of *pRD29A-LUC* and that the mutation affects *ROS1* expression.

### PKL is required for proper methylation of RdDM target loci

**Fig. 3:**
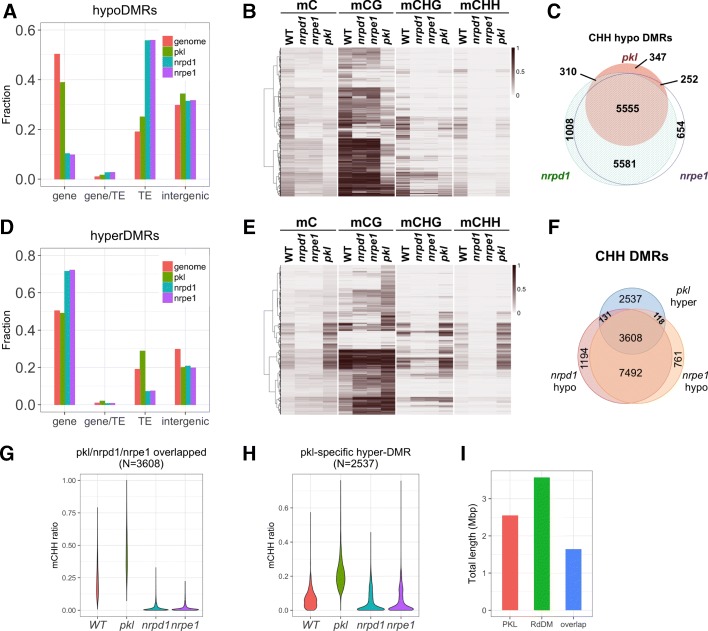
PKL affects DNA methylation levels at RdDM target loci. **a** Distribution of hypo differentially methylated regions (hypoDMRs) on genomic features. The Arabidopsis genome (TAIR10) was divided into four non-overlapping features based on the genome annotation. “gene/TE” represents genomics regions annotated as both genes and TEs. **b** *Heatmap* showing the DNA methylation levels at hypoDMRs identified in *pkl*. **c** Overlaps among CHH hypoDMRs identified in *pkl*, *nrpd1*, and *nrpe1*. The size of the *circle* is proportional to the number of DMRs identified in each mutant. **d** Distribution of hyperDMRs on the four non-overlapping genomic features. **e** *Heatmap* of the DNA methylation levels at hyperDMRs identified in *pkl*. **f** Overlaps among CHH hyperDMRs identified in *pkl* and CHH hypoDMRs identified in *nrpd1* or *nrpe1*. **g** *Violin plot* showing the distribution of CHH methylation levels at the 3608 *pkl* hyperDMR regions that are also identified as hypoDMRs of *nrpd1* and *nrpe1* (Fig. 3f). **h** *Violin plot* showing the distribution of CHH methylation levels at the 2537 *pkl-*specific hyperDMR regions (Fig. 3f). **i** Total lengths of mCHH DMRs identified in the *pkl* mutant (PKL), the *nrpd1* and *nrpe1* mutants (RdDM), and the overlapped regions between the two

To gain a full picture of the effect of PKL on DNA methylation, we performed whole-genome bisulfite sequencing using 14-day-old *pkl-1* seedlings (Col ecotype). Mutants of two core components of the RdDM pathway, *nrpd1-3* and *nrpe1-11*, were included as controls. *NRPD1* and *NRPE1*, respectively, encode the largest subunits of RNA polymerase IV and V. By comparing to the WT control, we identified 2641, 7265, and 6948 hypo differentially methylated regions (hypoDMRs) in *pkl*, *nrpd1*, and *nrpe1*, respectively. The average size of *pkl* hypoDMRs is smaller than those of *nrpd1* and *nrpe1* hypoDMRs (315 versus 436 and 433). Most of the hypoDMRs identified in *nrpd1* or *nrpe1* located to transposable elements (TEs) while hypoDMRs identified in *pkl* located more evenly to genes, TEs, and intergenic regions (Fig. 3a). Examination of the *pkl* hypoDMRs in the genome browser revealed loci where only non-CG methylation were reduced, as well as loci where DNA methylation was lost in all sequence contexts (Additional file 1: Figure S3A). Indeed, heatmap illustration of DNA methylation levels in all the 2641 *pkl* hypoDMRs indicated that both CG and non-CG methylation were reduced but rarely eliminated in the *pkl* mutant, while mutations in Pol IV or Pol V (*nrpd1* or *nrpe1*) resulted in elimination of CHH methylation and severe reduction of CHG methylation at most loci (Fig. 3b and Additional file 1: Figure S3B), indicating that RdDM activity is required to maintain CHH methylation at those regions. We thus analyzed the CHH methylation specifically [56]. We identified 12,394 and 12,010 hypomethylated CHH regions in *nrpd1* and *nrpe1*, respectively, and 11,136 (94.4%) were shared between the two mutants (Fig. 3c). Among the 6670 regions that showed significant reduction in CHH methylation in the *pkl* mutant, 91.7% (6117/6670) of them were also identified in *nrpd1* or *nrpe1* (Fig. 3c), indicating the majority of CHH hypoDMRs of *pkl* are RdDM targets. In most of the regions decreases in CHH methylation in *pkl* were not as dramatic as in *nrpd1* or *nrpe1*, but the 347 *pkl* unique regions exhibited significantly lower CHH methylation levels in *pkl* compared to *nrpd1* or *nrpe1* (Additional file 1: Figure S3C).

We also identified 4210 hyperDMRs in the *pkl* mutant, a higher number than the 2493 and 2715 hyperDMRs identified in *nrpd1* and *nrpe1*, respectively. HyperDMRs of *nrpd1* and *nrpe1* showed preferences for genes instead of TEs or intergenic regions, while hyperDMRs of *pkl* were more likely distributed to TEs, but not intergenic regions (Fig. 3d). We observed increases of DNA methylation levels in CG, CHG and CHH contexts in *pkl* hyperDMRs (Fig. 3e and Additional file 1: Figure S3B). However non-CG methylation was dependent on *NRPD1* or *NRPE1* in most of those regions (Fig. 3e and Additional file 1: Figure S3B), suggesting that the majority of hyperDMRs identified in *pkl* are also RdDM loci. Most of the *pkl* hyperDMRs already contained low levels of DNA methylation in WT plants (Fig. 3e). This was confirmed by visual inspection of the *pkl* hyperDMRs in the genome browser (Additional file 1: Figure S3D). More than 56% (3608/6394) of the CHH hyperDMRs identified in *pkl* overlapped with the CHH hypoDMRs of *nrpd1* and *nrpe1* (Fig. 3f). Compared to the WT, in the 3608 *pkl*/*nrpd1*/*nrpe1* overlapped regions, CHH methylation decreased to basal levels in *nrpd1* and *nrpe1*, whereas the methylation was significantly higher in *pkl* (Fig. 3g). In the 2537 *pkl*-specific regions, we also observed a decrease of CHH methylation in *nrpd1* and *nrpe1* (Fig. 3h). Those regions were not identified as hypoDMRs in *nrpd1* or *nrpe1* because in WT plants the majority of those regions had significantly lower CHH methylation levels than the overlapped regions (Fig. 3g; see Methods). Thus, the results indicated that the majority of differentially methylated regions of *pkl*, whether with increased or decreased DNA methylation, are RdDM target loci.

The total length of CHH DMRs of *pkl* added up to 2.55 Mbp, whereas the total length of RdDM loci, defined by CHH DMRs identified in both *nrpd1* and *nrpe1*, was 3.57 Mbp (Fig. 3i). The overlap between the two was 1.64 Mbp, indicating that at least 46% of the RdDM loci were affected by *PKL*. Overall the results above demonstrated that *PKL* is an important factor that is required to maintain the correct methylation pattern in roughly half of the genomic regions regulated by RdDM.

### PKL affects genome-wide 24-nt siRNA levels

We next tested whether PKL could influence DNA methylation by affecting 24-nt siRNA levels. First, we used northern blotting to examine the 24-nt siRNAs generated from the *RD29A* promoter. While *pRD29A* specific siRNAs were undetectable in the *ros1-1 nrpd1* mutant, their levels in *ros1-1 rdm18-1* and *ros1-1 rdm18-2* were comparable to those in WT and *ros1-1* plants (Additional file 1: Figure S4A), indicating that *RDM18/PKL* is not required for siRNA accumulation at the *RD29A* promoter.

We also examined siRNA levels at other endogenous RdDM loci using small RNA northern blotting. The methylation level at *AtSN1* was dependent on *RDM18*/*PKL* (Additional file 1: Figure S2B) and we also found a decrease in siRNA levels at this locus in the *ros1-1 rdm18-2* mutant (Additional file 1: Figure S4B). However, for another locus, *soloLTR*, where DNA methylation level also decreased in *pkl*, no changes in siRNA levels were observed (Additional file 1: Figure S4B). For the other two loci where we did not detect changes in DNA methylation levels in *rdm18*, *AtMu1*, and *siRNA1003*, no significant changes in siRNA levels were detected either (Additional file 1: Figure S4B), though siRNA levels did decrease in the *ros1-1 nrpe1* plants. As a control, neither *tasiRNA255* nor *miRNA171* was affected by the *rdm18* or *nrpe1* mutation (Additional file 1: Figure S4B).

**Fig. 4:**
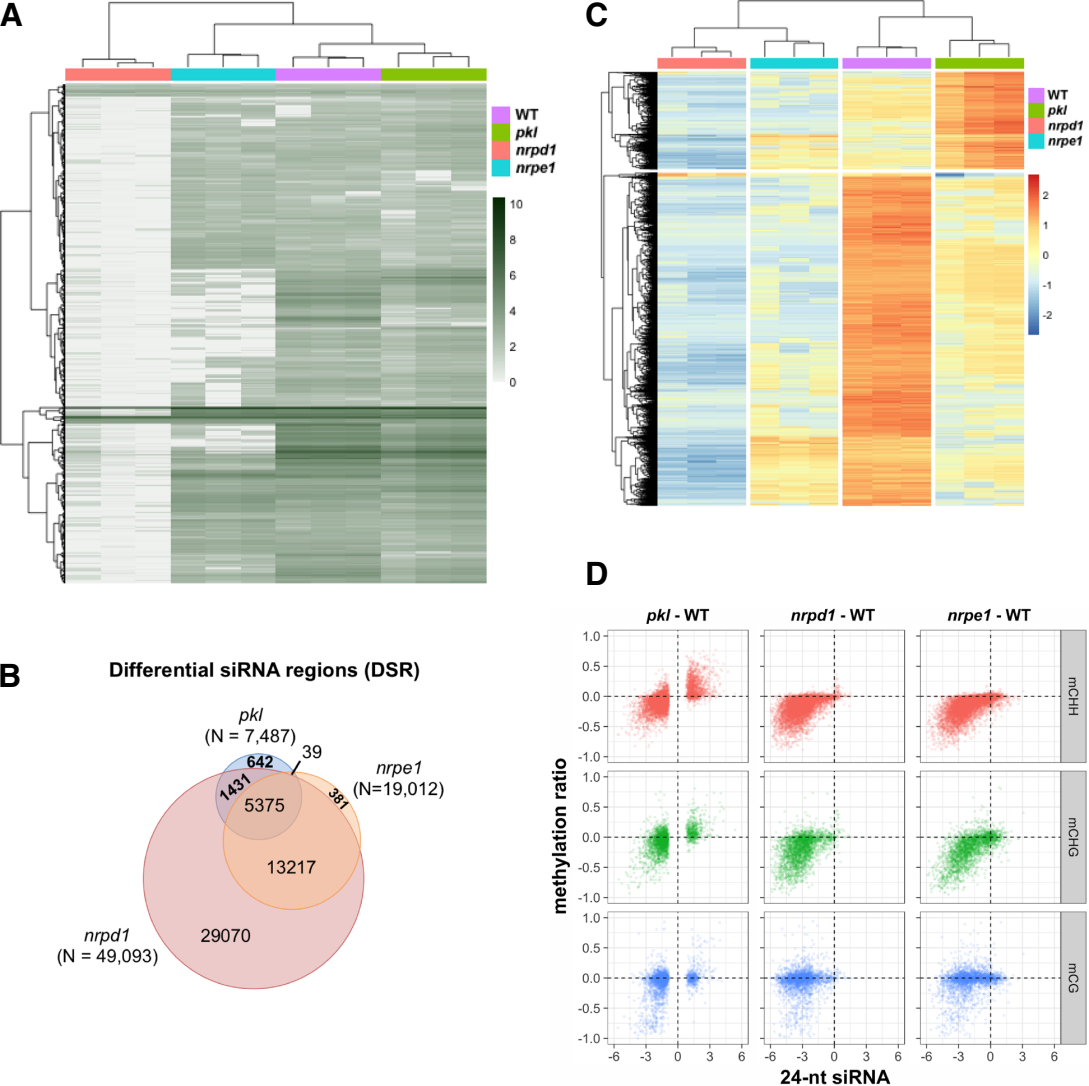
Effects of the *pkl* mutant on 24-nt siRNA abundance. **a** *Heatmap* showing the log(RPTM) value of 24-nt siRNAs in the genome. **b** Overlaps among differential siRNA regions (DSRs) identified in *pkl*, *nrpd1* and *nrpe1*. Both upregulated and downregulated DSRs are included. **c** *Heatmap* showing the relative abundance of 24-nt siRNAs at DSRs identified in *pkl*. **d** The relationship between siRNA level changes and DNA methylation level changes at DSRs identified in *pkl.* The difference in log(RPTM) values between the indicated mutant and WT were plotted on the *x-axis* and the difference in DNA methylation values were plotted on the *y-axis*

We next performed small RNA sequencing to understand the genome-wide changes in siRNA levels in the *pkl* mutant. We identified 57,094 regions where 24-nt siRNAs are expressed in either WT or mutant plants. As illustrated by the heatmap, the whole-genome profile of 24-nt siRNAs of the *pkl* mutant was more similar to WT than to *nrpd1* or *nrpe1* (Fig. 4a). While mutation of *NRPD1* eliminated siRNAs from most of the loci, PKL rarely reduced siRNAs to basal levels (Fig. 4a). It was reported that AGO4 protein level decreases significantly in mutants that are defective in siRNA production, presumably because formation of the siRNA-AGO4 complex stabilizes both the siRNA and AGO4 protein [57]. We thus examined AGO4 protein levels in the *pkl* mutant. Consistent with the less affected total siRNA abundance in *pkl* and *nrpe1*, anti-AGO4 western blot revealed no decreases of AGO4 proteins levels in *pkl-1*, *nrpe1-11*, or *pkl-1 nrpe1-11* plants, whereas mutation of *NRPD1* lead to significant reduction of AGO4 proteins (Additional file 1: Figure S4C).

Using a false discovery rate (FDR) cutoff of 0.01, 7487 differential siRNA regions (DSRs) were identified in the *pkl* mutant (Fig. 4b). More than 91% of the *pkl* DSRs overlapped with DSRs identified in *nrpd1* (Fig. 4b), in which 24-nt siRNAs at those regions decreased to basal levels (Fig. 4c). In contrary to *nrpd1* or *nrpe1*, whose DSRs are mainly hypoDSRs, a large number of hyperDSRs (n = 1691) was identified in *pkl* (Fig. 4c). Those regions contained medium levels of 24-nt siRNAs in WT and basal levels of siRNAs in *nrpd1*, indicating that they are normal RdDM targets. Interestingly more than 70% of the DSRs (5375/7487) identified in *pkl* were also affected by *NRPE1* (Fig. 4b), mutation of which led to reduction of siRNA levels in those regions (Fig. 4c). Despite the significantly smaller number of DSRs identified in *pkl* compared to *nrpe1* (7487 versus 19,012), 24-nt siRNAs also decreased in *pkl* at the majority of *nrpe1* affected regions (Additional file 1: Figure S4D), indicating that *PKL* and *NRPE1* tend to affect siRNA production at similar genomic loci.

We further explored the relationship between changes in 24-nt siRNA levels and DNA methylation levels in the *pkl* mutant. In most *pkl* DSR regions, increases and decreases in 24-nt siRNAs positively correlated with increases and decreases in non-CG methylation levels (Fig. 4d). In the same regions, *nrpd1* and *nrpe1* showed associated reduction in both siRNA and DNA methylation levels, with *nrpd1* having a stronger effect on siRNA reduction (Fig. 4d). Most of the CG methylation changes centered around zero, no matter decreases or increases in 24-nt siRNA levels were observed (Fig. 4d). These results indicated that mutation of *PKL* changed the abundance of 24-nt siRNAs at the affected RdDM loci, the levels of which correlated with non-CG methylation levels.

### PKL is required for Pol V function

**Fig. 5:**
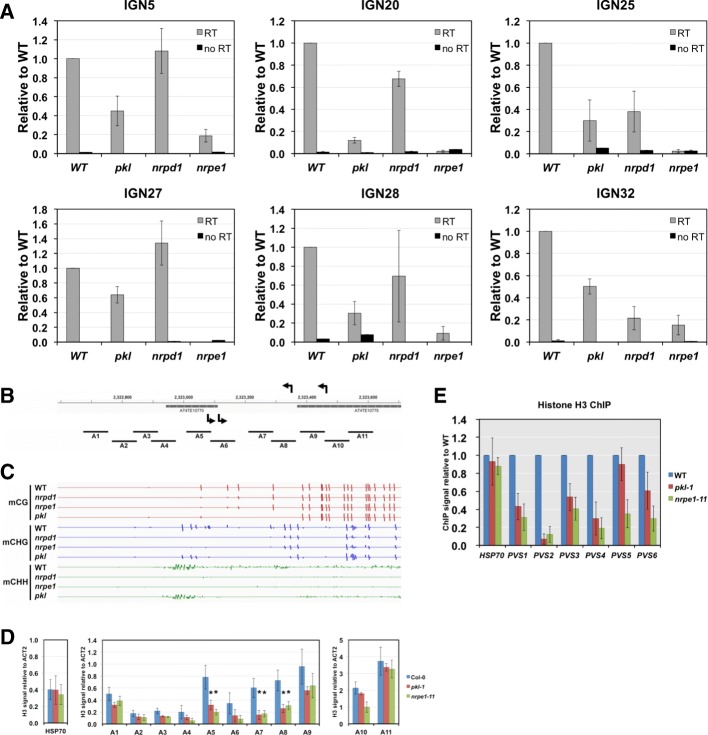
PKL is required for RNA Pol V-dependent noncoding RNA accumulation and nucleosome occupancy. **a** Non-coding RNA levels at six IGN loci were examined by real-time PCR. No RT (reverse transcriptase) samples serve as controls for genomic DNA contamination. All the transcript levels are shown on a relative scale with the level in WT (Col-0) plants being set to one. *Error bars* represent standard deviations calculated from three biological replicates. **b** *Diagram* showing the IGN5 locus on chromosome 4. *Arrows* above and below the coordinates indicate the position and direction where Pol V-dependent transcripts start. Positions of amplicons used for assaying nucleosome density in (**d**) were indicated by *black lines* labeled as A1 through A11. **c** A *screen shot* of IGV (Integrative Genomics Viewer) showing DNA methylation levels at the IGN5 locus. The *colored bars* (*red*, *blue*, *green*) represent the methylation levels of specific cytosines on the DNA double strands on a scale from –1 to 1; minus values indicate the methylated cytosine is on the reverse strand. **d** Nucleosome densities at the IGN5 locus assayed by anti-histone H3 ChIP. *Error bars* indicate standard deviations calculated from three biological replicates. All the signals are normalized to the ACT2 + 1 nucleosome; stars indicate *p* < 0.05 between the mutant and WT (Col-0) based on two-tailed t-tests. **e** PKL affects the positioning of Pol V-stabilized nucleosomes (PVS). Nucleosome positioning was examined by histone H3 ChIP following micrococcal nuclease digestion of the chromatin. The +1 nucleosome at *HSP70* served as a negative control. *Error bars* represent standard deviation calculated from three biological replicates

Based on the strong overlap between *PKL*-affected and *NRPE1*-affected siRNA regions, we tested whether *PKL* is required for the proper function of Pol V. We first examined the accumulation of Pol V-dependent transcripts. We randomly selected intergenic regions where Pol V-dependent transcripts can be detected using real-time PCR in previous studies [29]. Six regions with significantly decreased Pol V-dependent transcripts levels in the *pkl* mutant were identified (Fig. 5a). While scaffold RNAs generated by Pol V can be readily detected in WT plants, they were dramatically decreased to background levels in the *nrpe1* mutant (Fig. 5a). In general, the reduction of Pol V-dependent transcripts in *pkl* was not as dramatic as in *nrpe1* (Fig. 5a). Correspondingly, we observed a reduction of non-CG methylation at all six IGN loci in the *pkl* mutant (Additional file 1: Figure S5). We also observed reduction of Pol V dependent RNAs in the *nrpd1* mutant at IGN25 and IGN32, suggesting that their accumulation could be affected by DNA methylation levels (Additional file 1: Figure S5).

In order to further understand the effect of PKL at Pol V transcribed regions, we examined nucleosome densities at the IGN5 locus in the *pkl* mutant. The IGN5 locus is surrounded by two transposable elements and Pol V transcripts start from near the 3’ and 5’ end of the two TEs, respectively, and run in opposite directions [58] (Fig. 5b). A recent whole-genome study on Pol V transcripts also indicated that the IGN5 transcripts could start from inside the two TEs [59] (Fig. 5b). CHH methylation was decreased in *pkl* while abolished in *nrpe1* in this region (Fig. 5c). We examined nucleosome density within and around IGN5 using 11 primer pairs (Fig. 5b) and found that Pol V is required to promote nucleosome occupancy across the whole region except at the two ends, A1 and A11. Pol V has stronger effect of nucleosome stabilization in regions from A5 to A8, where Pol V presumably transcribes both strands (Fig. 5d). Except at A10, the effect of PKL on nucleosome occupancy in this region largely resembled that of Pol V (Fig. 5d), even though the *pkl* mutant had a milder effect on DNA methylation.

The scaffold RNAs generated by Pol V were shown to recruit SWI/SNF chromatin remodeling complexes that mediated nucleosome positioning at RdDM target regions [29]. We examined the effect of *PKL* on Pol V stabilized nucleosomes since PKL was shown to have nucleosome positioning activities in vitro [32]. We performed histone H3 ChIP following micrococcal nuclease digestion of the chromatin. Out of the six randomly chosen Pol V-stabilized nucleosomes, five exhibited significantly reduced occupancy in *pkl*, except for PVS5 (Fig. 5e). Similar to what we observed at IGN5, the nucleosome density signals in the *pkl* mutant were not statistically different from those in *nrpe1* at the 5 affected loci (Fig. 5e).

### The effect of *pkl* on gene and *TE* silencing

In order to further understand the function of PKL in gene and TE silencing, a messenger RNA (mRNA)-seq experiment was performed in two-week-old *pkl-1* seedlings, as well as in the two RdDM mutants *nrpd1-3* and *nrpe1-11*. Statistical testing using a FDR cutoff of 0.05 and fold change cutoff of 2 identified 25 transposable elements (TEs) and 651 genes that were differentially expressed in the *pkl* mutant (Additional file 1: Figure S6A). The majority of DEGs (differentially expressed genes) of *pkl* did not show an expression change in *nrpd1* or *nrpe1* (Additional file 1: Figure S6A). Eighteen of 274 upregulated genes and six derepressed TEs of *pkl* also showed increased expression in *nrpd1* or *nrpe1* (Additional file 1: Figure S6B and S6C); similarly, 17 of 377 downregulated genes of *pkl* also showed decreased expression in *nrpd1* or *nrpe1* (Additional file 1: Figure S6D). Consistent with a previous report, in the *pkl* mutant 34% (*n* = 92) of the upregulated genes and 42% (*n* = 159) of the downregulated genes were also targets of H3K27me3, an epigenetic modification important for the silencing of developmentally regulated genes [46, 60]. The differences in the number of differentially expressed genes (DEGs) between *pkl* and *nrpd1*/*nrpe1* are consistent with the role of *PKL* in developmental regulation and a role of RdDM in TE methylation.

**Fig. 6:**
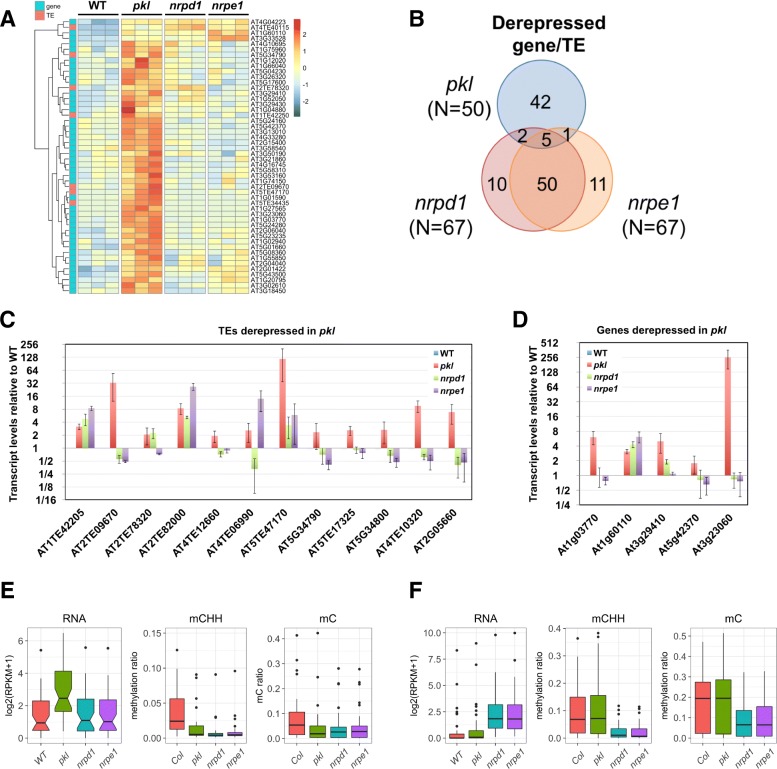
The effects of PKL on the silencing of genes and TEs. **a** *Heatmap* showing the relative transcript levels of the 50 derepressed genes/TEs in *pkl* that overlapped with hypo DMRs. **b** Overlaps among derepressed genes/TEs identified in *pkl*, *nrpd1*, and *nrpe1* that overlapped with their respective hypo DMRs. **c** qRT-PCR verification of 12 upregulated transposable elements identified in *pkl*. **d** qRT-PCR verification of five upregulated genes identified in *pkl*. Transcript levels relative to WT were shown. *Error bars* represent standard deviations of three biological replicates. **e** *Boxplots* of the mRNA and DNA methylation levels of the 42 genes/TEs that are derepressed in *pkl* but not in RdDM mutants as shown in (**b**). **f** *Boxplots* of the mRNA and DNA methylation levels at the promoter region of the 50 genes/TEs that are derepressed in both *nrpd1* and *nrpe1* but not in *pkl* as shown in (**b**)

We found that 50 of the 296 upregulated genes/TEs in *pkl* overlapped with 52 hypoDMRs within 1-kb regions upstream and downstream of the gene/TE body. However, expression of most of the 50 genes/TEs did not show a significant increase in *nrpd1* or *nrpe1* (Fig. 6a). Indeed, the majority of upregulated genes/TEs in *nrpd1* and *nrpe1* were associated with hypoDMRs (67 out of 90 for *nrpd1* and 67 out of 81 for *nrpe1*), but only eight were shared between *nrpd1/nrpe1* and *pkl* (Fig. 6b).

**Fig. 7:**
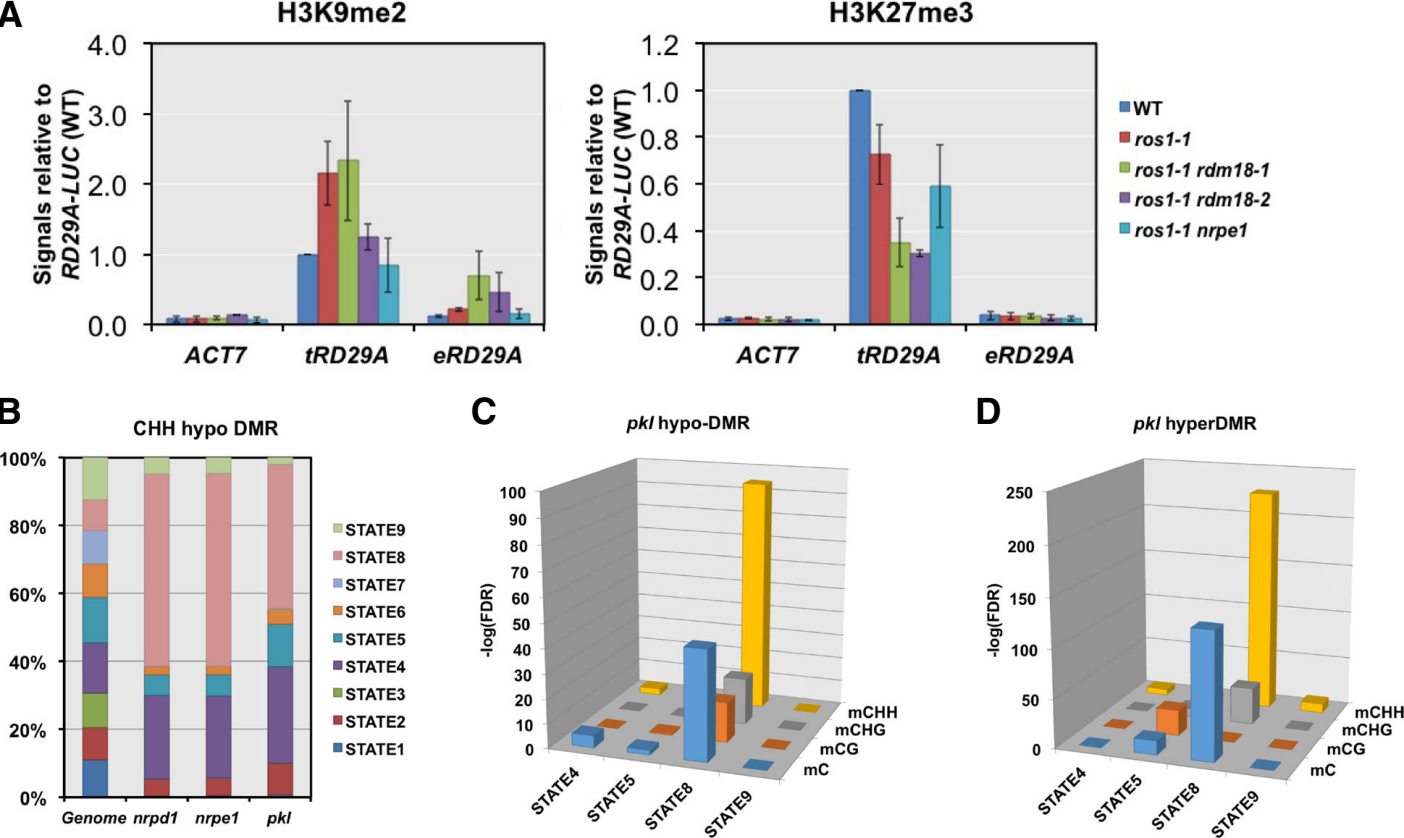
The correlation between PKL affected loci and repressive histone modifications. **a** H3K9me2 and H3K27me3 levels at the transgenic and endogenous *RD29A* (*tRD29A* and *eRD29A*) promoter measured by the chromatin immunoprecipitation (ChIP) assay. The *ACT7* promoter (*ACT7*) serves as a negative control for the two repressive histone modifications. The ChIP DNA was quantified using real-time PCR and normalized to the signal at *tRD29A* in WT plants. *Error bars* represent standard deviations calculated from three biological replicates. **b** Distribution of nine different chromatin states on the whole genome or the CHH hypo-DMRs of the three mutants (*nrpd1-3*, *nrpe1-11*, and *pkl-1*). **c**, **d** Log transformed FDR values (–log10) of the overlap between hypoDMRs (**c**) and hyperDMRs (**d**) identified in *pkl* and the four repressive chromatin states

Most of the derepressed genes and TEs had low expression levels in WT plants. We thus performed qRT-PCR to confirm their upregulated expression in *pkl*. Among the 17 randomly selected genes/TEs (12 TEs and five genes), 15 were confirmed to exhibit significantly increased expression in the *pkl* mutant (Fig. 6c and d). Among the qRT-PCR verified genes/TES, two TEs (AT1TE42205 and AT2TE82000) and one gene (AT1G60110) were also identified as derepressed in *nrpd1* and *nrpe1* (Fig. 6c and d), suggesting that decreased DNA methylation may be responsible for their derepression.

We further analyzed the expression and DNA methylation levels of the 42 and 50 genes/TEs that are specifically affected by PKL and RdDM (Fig. 6b). Transcript levels of the 42 *pkl* affected genes/TEs in *nrpd1*/*nrpe1* were very similar to WT plants (Fig. 6e). Consistent with the observation that the majority of *pkl* DMRs were RdDM loci, the DNA methylation level, especially CHH methylation level, decreased in the promoter region of the 42 affected genes/TEs in *nrpd1* and *nrpe1* (Fig. 6e), suggesting that decreased DNA methylation are not sufficient to release silencing at those genes/TEs. In contrast, DNA methylation levels at the 50 RdDM affected loci were significantly higher than the *pkl* affected genes/TEs and decreased DNA methylation was correlated with increased transcript levels in *nrpd1* and *nrpe1* (Fig. 6f). Changes in the transcript level or the DNA methylation level at the 50 RdDM affected loci were not observed in *pkl* (Fig. 6f).

Among the 25 differentially expressed TEs in *pkl*, 22 exhibited increased transcript levels (Additional file 1: Figure S6A), consistent with a role of PKL in transcriptional silencing of some TEs. The number of TEs that were derepressed in *nrpd1* and *nrpe1* were 44 and 42, respectively, and 36 of them were shared between the two (Additional file 1: Figure S6C). The *pkl* mutant shared six derepressed TEs with *nrpd1* or *nrpe1* (Additional file 1: Figure S6C). The 16 TEs derepressed in *pkl* but not in *nrpd1* or *nrpe1* exhibited slightly reduced DNA methylation in *pkl* (Additional file 1: Figure S6E). However, similar or stronger decreases in DNA methylation levels were also observed in *nrpd1* and *nrpe1* (Additional file 1: Figure S6E). This was in contrast to the 32 TEs that were derepressed in *nrpd1* and *nrpe1* but not *pkl* (Additional file 1: Figure S6C), where the correlation between decreased DNA methylation and increased transcripts was clear (Additional file 1: Figure S6F). These results suggest that PKL also has a role in transcriptional silencing that is independent of DNA methylation.

### The relationship between PKL and repressive histone modifications

We next tested if other repressive epigenetic modifications besides DNA methylation could be involved in transcriptional silencing mediated by *PKL*. One of the mechanisms by which PKL represses gene expression is by promoting H3K27me3 deposition [45, 46]. The level of H3K9me2 is tightly linked to non-CG DNA methylation [13]. We wonder if repressive histone modifications such as H3K27me3 and H3K9me2 were also involved in silencing at the transgenic *RD29A* promoter. Indeed, substantial levels of H3K27me3 and H3K9me2 were detected in WT plants and significant decreases of H3K27me3 were observed at the transgenic *RD29A* promoter in the *ros1-1 rdm18* mutant (Fig. 7a).

A recent study using 16 features classified the Arabidopsis chromatin into nine different states, each represented by a specific combination of features [61]. Of those, states 4, 5, 8 and 9 are the ones that are associated with transcriptional repression [61]. We examined the distribution of DMRs identified in *pkl*, *nrpd1* and *nrpe1* over the nine states. While the total lengths of the nine artificial types of chromatin are similar in the genome, *nrpd1* and *nrpe1* CHH hypoDMRs showed a clear preference for state 4 and state 8 (Fig. 7b). Similar distribution over these chromatin states was observed for CHH hypoDMRs of *pkl* (Fig. 7b). The FDRs for any types of DMRs (mC, mCG, mCHG, or mCHH) of *pkl* to reside in state 8 by chance varied from 3 × 10^–95^ to 5 × 10^–17^ (Fig. 7c). This strong preference for state 8 chromatin was also observed for hypoDMRs identified in *nrpd1* and *nrpe1*, with even smaller FDR values (Additional file 1: Figure S6A). Interestingly, we observed the same pattern for hyperDMRs of *pkl* (Fig. 7c), consistent with our findings that both hypoDMRs and hyperDMRs of *pkl* are mainly RdDM target loci. State 8 chromatin is enriched for heterochromatic features including H3K9me2, H3K27me1, histone H3.1, and varying levels of H3K27me3 [61]. Considering that the reported roles of PKL in both promoting and antagonizing H3K27me3 [46] and that elimination of H3K27me1 does not have an effect on genome DNA methylation levels [62], we speculated that PKL may affect DNA methylation through its effect on H3K27me3. We examined DNA methylation levels in the H3K27me3-defective mutant *clf-29* [63]. CLF encodes a histone methyltransferase specific for histone H3 lysine 27 [64]. Compared to hypo-DMRs identified in *pkl*, much fewer DMRs were identified in the *clf-29* mutant (CHH hypo-DMR = 185; CHG hypo-DMR = 27; CG hypo-DMR = 489). Non-CG DNA methylation levels were also very similar between *clf-29* and WT plants at *pkl* CHH hypo-DMRs (Additional file 1: Figure S6C). These results indicate that decreased H3K27me3 levels in *clf-29* do not lead to changes in DNA methylation levels at PKL-affected regions.

## Discussion

### The effects of PKL at RdDM heterochromatin

In this study, we characterized the effects of PKL in RNA-directed DNA methylation. Mutation of *PKL* changed the DNA methylation pattern of about half of RdDM loci and affected the noncoding RNAs generated by RNA Pol V. We also found that *PKL* had both DNA methylation-dependent and methylation-independent roles in gene/TE silencing.

PKL is a CHD3-type chromatin remodeling factor that was shown to regulate many developmental processes [35–38, 46, 65, 66]. Its effect on DNA methylation was underappreciated because microarray-based transcriptome analyses of *pkl* failed to identify statistically significant overlap of differentially expressed genes with DNA methylation mutants [46]. Since TEs are underrepresented in the ATH1 microarray and their transcript levels are typically too low to be detected by the technology, the effect of PKL on TEs was largely unknown [45]. This study identified genome-wide changes in DNA methylation patterns in the *pkl* mutant and found that the DNA methylation changes were predominantly at RdDM loci. RdDM mainly targets TEs and loss of RdDM activity does not lead to dramatic changes in the expression level of genes (Fig. 3a) [11]. Thus, our results are consistent with previous findings and revealed a new role of PKL in modulating DNA methylation at RdDM loci.

The involvement of *PKL* in regulating DNA methylation levels of RdDM loci is supported by several pieces of evidence. First, PKL was identified in the *ros1* suppressor screen and is required to promote non-CG methylation and transcriptional silencing of the *pRD29A-LUC* transgene, which requires RNA-directed DNA methylation for silencing (Fig. 1d and Additional file 1: Figure S2A). Tens of components involved in RdDM have been identified from the same screen [50]. Second, the transcript level of the DNA demethylase gene *ROS1* decreased in all the *rdm18/pkl* alleles. Independent studies found that the *ROS1* transcript level decreases in mutants defective in DNA methylation or in plants treated with DNA methylation inhibitors [53, 55, 67, 68]. Two recent studies identified a TE-derived *cis*-regulatory element, DNA methylation of which positively regulates *ROS1* expression [54, 55]. Mutation of *PKL* reduced the DNA methylation level at the same *cis*-element. Third, whole-genome bisulfite sequencing analyses indicated that PKL was required for proper methylation of about half of the RdDM target loci (Fig. 3 and Additional file 1: Figure S3). It remains to be tested if further mutation of other CHD family chromatin remodelers could enhance the DNA methylation phenotype of *pkl* [32].

Correlated with changes in DNA methylation levels, significant changes in the 24-nt siRNA profile of *pkl* were also observed (Fig. 4). The numbers of hyperDSRs identified in *pkl* were significantly smaller than the numbers of CHH hyperDMRs. This may be due to the technical limitation of the small RNA sequencing experiment. Regions that showed increases in DNA methylation and siRNA levels in *pkl* were RdDM target loci that contain low levels of DNA methylation and 24-nt siRNAs (Figs. 3e and 4c). While whole-genome bisulfite sequencing covers the genome relatively evenly, small RNA reads are dominated by loci that are more highly methylated. Thus, deeper sequencing may be needed to reveal the changes in siRNA levels at the other hyperDMRs. It remains to be determined as to how PKL affects siRNA production. The majority of 24-nt DSRs identified in *pkl*, whether increased or decreased, are also affected by *nrpe1* (Fig. 4b), suggesting that PKL may affect secondary siRNA production as NRPE1 does. Other evidence also suggests that the function of Pol V was affected by *pkl*. Correlated with DNA methylation decreases, the scaffold RNAs generated by Pol V and the occupancy of Pol V stabilized nucleosomes were reduced in the *pkl* mutant (Fig. 5a and c).

RNA-seq analyses identified 50 genes/TEs that were derepressed and correlated with decreases in DNA methylation in *pkl*. However similar analyses in *nrpd1* and *nrpe1* indicated that decreased DNA methylation was not sufficient to cause derepression for the majority of them (Fig. 6). Overall these results demonstrated that multiple aspects of RNA-directed DNA methylation were affected in the *pkl* mutant.

### Possible functions of PKL at RdDM target loci

ATP-dependent chromatin remodeling factors are conserved helicase-derived machineries that are involved in almost all aspects of chromatin regulation [20]. Arabidopsis contains 45 ATP-dependent chromatin remodeling factors, out of which DDM1 and PKL are the only two that were confirmed to exhibit nucleosome remodeling activity in vitro [32, 69]. It is believed that DDM1 promotes CMT2-dependent CHH methylation in the middle of long transposable elements by allowing CMT2 to better access its substrate DNA [12]. The observation that PKL is required for both promoting and repressing DNA methylation at RdDM loci is different from other known RdDM mutants, suggesting that the phenotype is not through affecting the expression of any single component of the RdDM pathway. Indeed, transcriptome analyses in *pkl-1* did not identify reduced expression in any known RdDM component genes (Additional file 2: Table S4). It was shown before that PKL could bind to certain TEs [45]. The animal homologs of PKL, Mi-2α/β, were also recruited to the heterochromatin by MeCP2 (methyl CpG binding protein 2) [70]. We propose that PKL binds to the chromatin of RdDM target loci and affects DNA methylation through its nucleosome remodeling activity.

PKL could affect RNA-directed DNA methylation by regulating nucleosome positioning. Similar to other classic chromatin remodelers, PKL exhibits nucleosome “sliding” activity in vitro [32]. In Arabidopsis, nucleosome-bound DNA exhibits higher methylation levels than nucleosome-free DNA and non-CG methylation are promoted by the histone modification H3K9me1/2 [13, 71]. Thus, simply changing the nucleosome positioning could change DNA methylation patterns. Indeed, the positioning of several Pol V stabilized nucleosomes was altered in the *pkl* mutant (Fig. 5e). Alternatively, PKL may function in regulating the nucleosome conformation, which in turn has an effect on noncoding RNA production by Pol IV and Pol V or on the activity of DNA methyltransferases. We found that in addition to non-CG methylation, CG methylation was also affected at some DMRs identified in *pkl* (Fig. 3b and e), suggesting that the activity of other DNA methyltransferases, in addition to DRM2, was affected in those regions.

PKL participates in the Pol V transcription process is another possibility by which PKL affects DNA methylation at RdDM loci. Immunoaffinity purification of Pol V identified the chromatin remodeler DRD1, but not PKL [15, 23, 27, 28]. Though the specific activity of DRD1 in promoting Pol V function is unknown, DRD1 is required for the association of Pol V to the chromatin and its mutant has a similar DNA methylation profile as *nrpe1* [24, 56]. Despite its functional importance, DRD1 is unlikely the only chromatin remodeler participating in the transcription process. In animals, different CHD proteins are required at the initiation, elongation, or termination phase of Pol II transcription [72, 73], indicating that their heterogeneous biochemical activities suit multiple aspects of the transcription cycle. The different effects of PKL and DRD1 on DNA methylation suggest they could function in different phases of Pol V transcription [56]. For example, CHD1 from yeast and Drosophila function during transcription elongation and facilitate reassembly and repositioning of nucleosomes after the polymerase [72, 74]. Although CHD remodelers can contribute to different aspects of transcription, they do not necessarily exhibit a strong association with Pol II. Interestingly, similar to CHD1, PKL primarily exists as a monomer in vivo [32, 75] and the *pkl* mutant exhibits reduced nucleosome occupancy at Pol V transcribed regions (Fig. 5). In addition, a number of genes/TEs that were derepressed in *pkl* were not due to decreases in DNA methylation (Fig. 6e), suggesting that a DNA-methylation independent role of PKL in promoting transcriptional silencing via its nucleosome remodeling activity. In the future, understanding the in vivo biochemical activity of PKL on the chromatin and its correlation with DNA methylation will be important.

## Conclusions

We found that the CHD3 protein PKL has an unexpected role in the regulation of DNA methylation levels at the loci targeted by RNA-directed DNA methylation. The changes in CHH methylation in *pkl* positively correlates with changes in Pol IV-dependent siRNAs and Pol V-dependent scaffold RNAs. These findings significantly advance our understanding of how RNA-directed DNA methylation can be regulated and highlight the diverse functions of CHD proteins in the regulation of chromatin activities.

## Methods

### Plant materials and growth conditions

Plants in the C24 ecotype (WT, *ros1-1*, *ros1-1 nrpd1*, *ros1-1 nrpe1*) carry a homozygous T-DNA insertion that contains the *pRD29A-LUC* and *p35S-NPTII* transgenes. For genetic screening, a T-DNA mutagenized and an EMS-mutagenized *ros1-1* populations were generated and screened for plants that show increased luciferase signals as described previously [50]. Plants were grown in growth chambers or air-conditioned rooms at 22 °C with 16 h-8 h light-dark cycle.

### DNA methylation analyses of individual loci

For southern blotting, genomic DNA was extracted from two-week-old Arabidopsis seedlings using the typical CTAB method. The genomic DNA was digested with a DNA methylation-sensitive restriction endonuclease (NEB) and 5 μg of the digested DNA was loaded into a 1% agarose gel and separated at 40 V for 12 h. Then southern blotting was performed following a standard protocol.

For individual bisulfite sequencing, genomic DNA was extracted from two-week-old seedlings using the Plant DNeasy Mini Kit (Qiagen). Then 2 μg of genomic DNA was subjected to sodium bisulfite treatment and purification using the EpiTect Plus Bisulfite Kit (Qiagen). Then locus-specific primers (Additional file 2: Table S5) were used to amplify regions of interested and the PCR product were cloned into the T-easy vector (Promega). At least 18 unique sequences from each genotype/locus was obtained and analyzed at the CyMATE website (http://www.cymate.org).

### RT-PCR

RT-PCR and qRT-PCR were performed as described previously [50]. Briefly Trizol reagent (Life Technologies) extracted total RNA was subjected to DNase I treatment (Ambion) and RT using Superscript III First Strand Synthesis Kit (Life Technologies). The synthesized complementary DNA (cDNA) was then diluted to 5–10 ng/μL and 5 μL was used for each RT-PCR or qRT-PCR reaction. For RT-PCR, the optimal PCR cycle number for each primer pair was empirically determined. The primers used for RT-PCR analyses are listed in the Supplementary Material (Additional file 2: Table S5).

### Analyses of Pol V-dependent transcripts

Detection of Pol V-dependent transcripts was performed by following a published protocol [29]. Briefly, total RNA was extracted from two-week-old seedlings using Plant RNeasy Mini Kit (Qiagen). The eluted RNA was treated with Turbo DNase I (Ambion) at 37 °C for 30 min. Then 2 μg of RNA without contaminated DNA was used for synthesis the first strand cDNA using SuperScript III First-Strand Synthesis System (Invitrogen) with the random hexamers. Then 200 ng of cDNA was used per real-time reaction using transcript-specific primers.

### Small RNA northern blotting

Northern blotting for small RNA analyses was performed as described previously [76]. Briefly, small RNAs were extracted using the TRIzol reagent and PEG precipitation and then separated on a 15% polyacrylamide gel at 200 V for 3–4 h. The small RNA was stained with ethium bromide and electro-transferred to the Hybond-N+ membrane (GE Lifesciences). Small RNA hybridization was carried out in PerfectHyb buffer (Sigma) overnight at 38 °C. Probes were produced by PCR amplification in the presence of [α-^32^P] dCTP. Primers used for generating the probes are listed in Additional file 2: Table S5.

### Whole-genome bisulfite sequencing and data analyses

Genomic DNA was extracted from two-week-old *pkl-1* seedlings using the Plant DNeasy mini kit (Qiagen) and sent to BGI (Shenzhen, China) for whole-genome bisulfite sequencing. For *clf-29* and the corresponding WT control, two-week-old seedlings were grown under the same conditions and sent to Core Facility for Genomics at Shanghai Center for Plant Stress Biology (PSC) for whole-genome bisulfite sequencing.

For the analyses of BS-seq data, first adapter sequences and low-quality reads (Q < 20) were trimmed and clean reads were mapped to the TAIR10 genome using BSMAP [77]. The method for the identification of total C DMRs (differentially methylated regions) was reported before [78]. For the identification of different types of DMRs (mCG, mCHG, and mCHH) we followed a published method [56]. Briefly, the genome was divided in 100-bp bins and CG, CHG, CHH methylation levels in each bin (covered at least four times) was calculated and compared between WT and mutant plants. Bins that show differences in DNA methylation levels (mCG > 0.4, mCHG > 0.2, mCHH > 0.1) were recorded and filtered based on Fisher’s exact test and multi-testing corrected *p* values (cutoff = 0.05). DMRs were generated by joining bins that are no more than 200 bp apart.

### Transcriptome sequencing and data analyses

Total RNAs were extracted from two-week-old WT, *pkl-1*, *nrpd1-3*, and *nrpe1-11* seedlings using Trizol reagent (Life Technologies). PolyT purification of messenger RNAs, stranded RNA library preparation and paired end sequencing were performed using Illumina reagents following the manufacturer’s instructions at Genomics Core Facility of PSC. For data analyses adapter sequences and low quality bases (q < 30) were trimmed and clean reads were mapped to the TAIR10 reference genome using the subread package. Read counts for both genes and TEs were produced using the featureCounts command and statistical testing was performed using the edgeR package in R [79].

### Small RNA sequencing and data analyses

Total RNA extracted from two-week-old WT, *pkl-1*, *nrpd1-3*, and *nrpe1-11* seedlings were separated on a PAGE gel and 18–30 nt fraction of the gel was cut for small RNA purification. Library preparation and sequencing were performed using Illumina reagents according to the manufacturer’s instructions at Genomics Core Facility of PSC. For data analyses, adapter sequences and low quality bases (q < 30) were trimmed and clean reads of size 18–30 nt were mapped to the TAIR10 genome after removing reads that can be mapped to annotated structural RNAs (rRNAs, tRNAs, snRNAs, and snoRNAs). Only uniquely mapped reads were used for downstream analyses. Read counts in every 100-bp bin of the genome were generated using bedtools coverage [80] and were normalized to reads per 10 million (RPTM) according to the total number of mapped reads (excluding structural sRNAs). Only bins with a normalized RPTM value of 24-nt sRNAs higher than 5 in any plant were retained for differential analyses using the edgeR package [79].

### Chromatin immunoprecipitation

Chromatin immunoprecipitation was performed according to a published protocol [46]. Typically, two-week-old seedlings grown on ½ MS plates supplemented with 1% sucrose were used as the starting material. After crosslinking and nuclei extraction, the chromatin was fragmented using either directly sonication or sonication after MNase digestion. Afterwards soluble fraction of fragmented chromatin was incubated with anti-H3 (Abcam ab1791), anti-H3K27me3 (Millipore 07-449) or anti-H3K9me2 (Abcam ab1220) antibodies for overnight at 4 °C. After washing and reverse crosslinking, the immunoprecipitated DNA was then purified using PCI extraction and examined using gene specific primers by qPCR.

### Western blotting

After total proteins were extracted, the proteins were heated at 95 °C for 5 min before being separated on SDS-PAGE for Commassie Blue staining. A standard western blotting protocol was used with anti-AGO4 antibody (Agrisera, AS09617) at 1:5000 dilutions.

## Additional files

Additional file 1: Figure S1. *RDM18*/*PKL* promotes silencing at the *p35S-NPTII* transgene but does not affect DNA methylation levels. **Figure S2.** *RDM18* is required for proper methylation at RdDM target loci. **Figure S3.** Characterization of differentially methylated regions in the *pkl* mutant. **Figure S4.** Effects of *pkl* on 24-nt siRNA levels and AGO4 protein levels. **Figure S5.** Analyses on TEs and genes that are differentially expressed in mutants. **Figure S6.** Chromatin features of the RdDM loci. (PDF 6385 kb)

Additional file 2: Table S1. Statistics of RNA-seq data. **Table S2.** List of differentially expressed genes in the *pkl* mutant identified by RNA-seq. **Table S3.** List of differentially expressed TEs in the *pkl* mutant identified by RNA-seq. **Table S4.** Transcript level of genes involved in DNA methylation regulation. **Table S5.** List of primers used in this study. (XLSX 218 kb)

## Abbreviations

DEG: Differentially expressed genes
DMR: Differentially methylation regions
ncRNA: Non-coding RNA
nt: Nucleotide
PCR: Polymerase chain reaction
Pol IV: RNA polymerase IV
Pol V: RNA polymerase V
RdDM: RNA-directed DNA methylation
siRNA: Small interfering RNA
