# Supplementary material for: The developmental regulator PKL is required to maintain correct DNA methylation patterns at RNA-directed DNA methylation loci
Source: Genome Biol. 2017 May 31;18:103. doi: 10.1186/s13059-017-1226-y (PMC5452414; doi:10.1186/s13059-017-1226-y)
Supplement: Supplementary file 1 — RDM18/PKL promotes silencing at the p35S-NPTII transgene but does not affect DNA methylation levels. Figure S2. RDM18 is required for proper methylation at RdDM target loci. Figure S3. Characterization of differentially methylated regions in the pkl mutant. Figure S4. Effects of pkl on 24-nt siRNA levels and AGO4 protein levels. Figure S5. Analyses on TEs and genes that are differentially expressed in mutants. Figure S6. Chromatin features of the RdDM loci. (PDF 6385 kb) [file 13059_2017_1226_MOESM1_ESM.pdf]

Figure S1

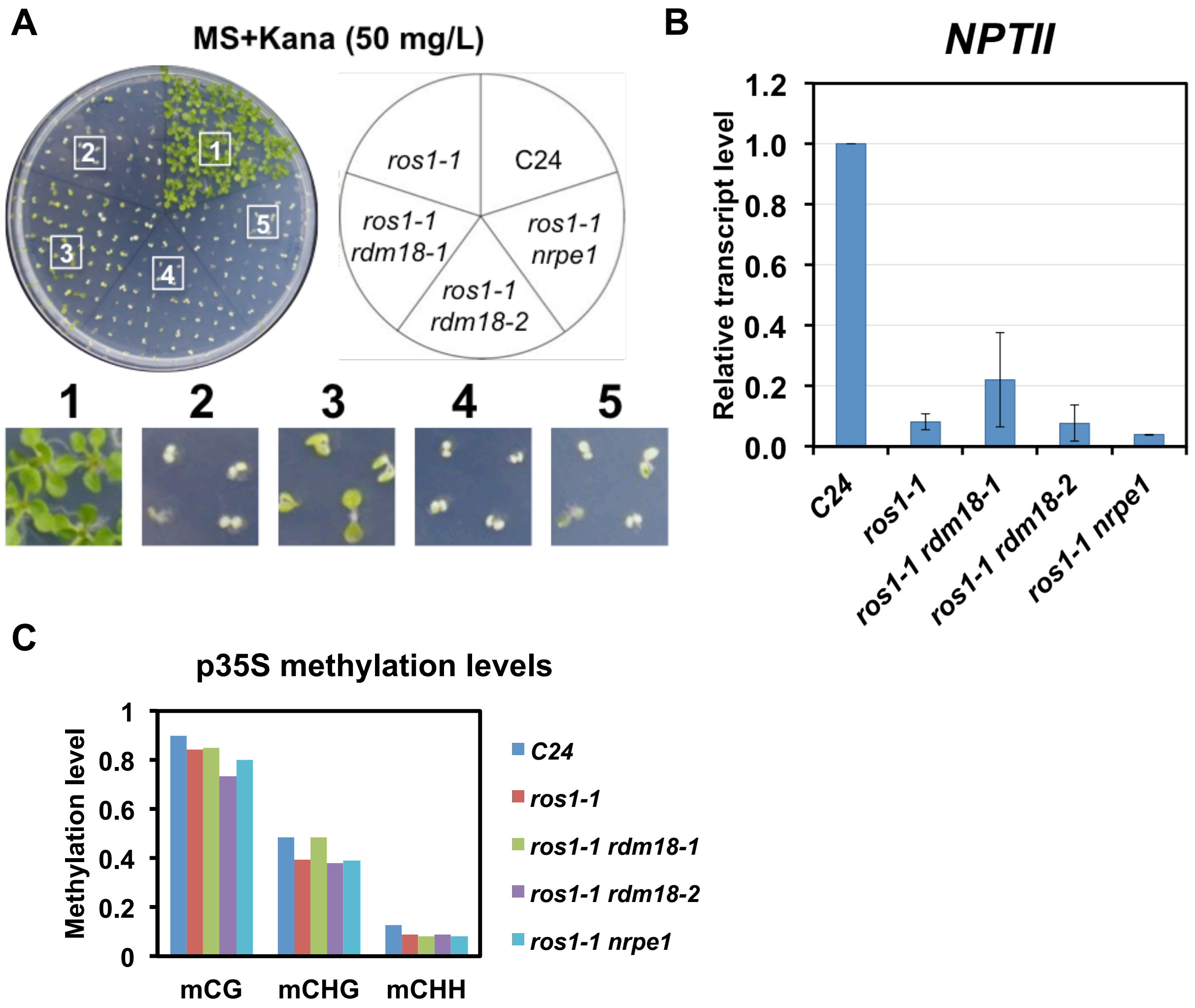

Figure S1. *RDM18*/*PKL* promotes silencing at the *p35S-NPT II* transgene but does not affect DNA methylation levels. (A) Kanamycin-resistance phenotype of 2-week-old seedlings. Genotypes of each part of the plate were indicated on the right circle. (B) Relative transcript level of the *NPT II* transgene in *rdm18* mutants. Error bars indicate standard deviations calculated from three biological replicates. (C) DNA methylation levels of the 35S promoter in *rdm18* seedlings were measured using individual bisulfite sequencing.

Figure S2

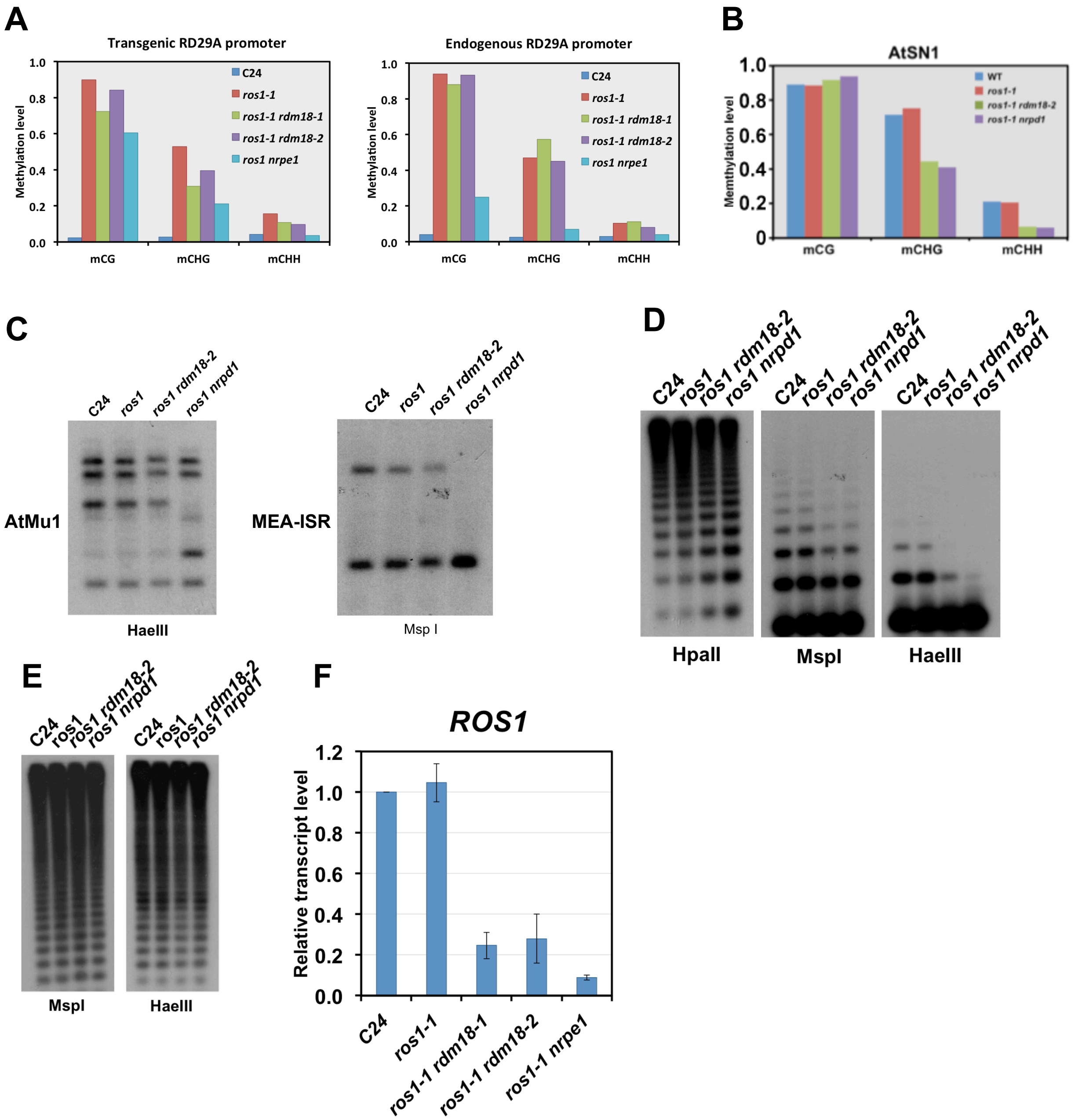

Figure S2. *RDM18* is required for proper methylation at RdDM target loci. (A and B) DNA methylation levels at the transgenic and endogenous *RD29A* promoter region (A) and AtSN1 (B) were measured by bisulfite sequencing using locus-specific primers. At least 18 independent colonies were sequenced for each sample. (C) DNA methylation levels at two typical RdDM loci (AtMu1 and MEA-ISR) were examined by southern blotting following digestion of the genomic DNA by methylation sensitive enzymes (HaeIII and MspI). Smaller fragments indicate loss of DNA methylation at the enzyme recognition site. (D and E) DNA methylation levels at the 5S rDNA repeats (D) and centromeric 180bp repeats (E) were examined using methylation-sensitive enzyme digestion followed by southern blotting. (F) The relative transcript level of the *ROS1* gene was examined by real-time PCR in *ros1 rdm18* double mutants. The transcript level in mutant plants were compared to the wild type plants (C24). Error bars represent standard deviations calculated from 2 biological replicates. The *ros1-1* mutation is a point mutation that does not change *ROS1* transcript levels.

Figure S3

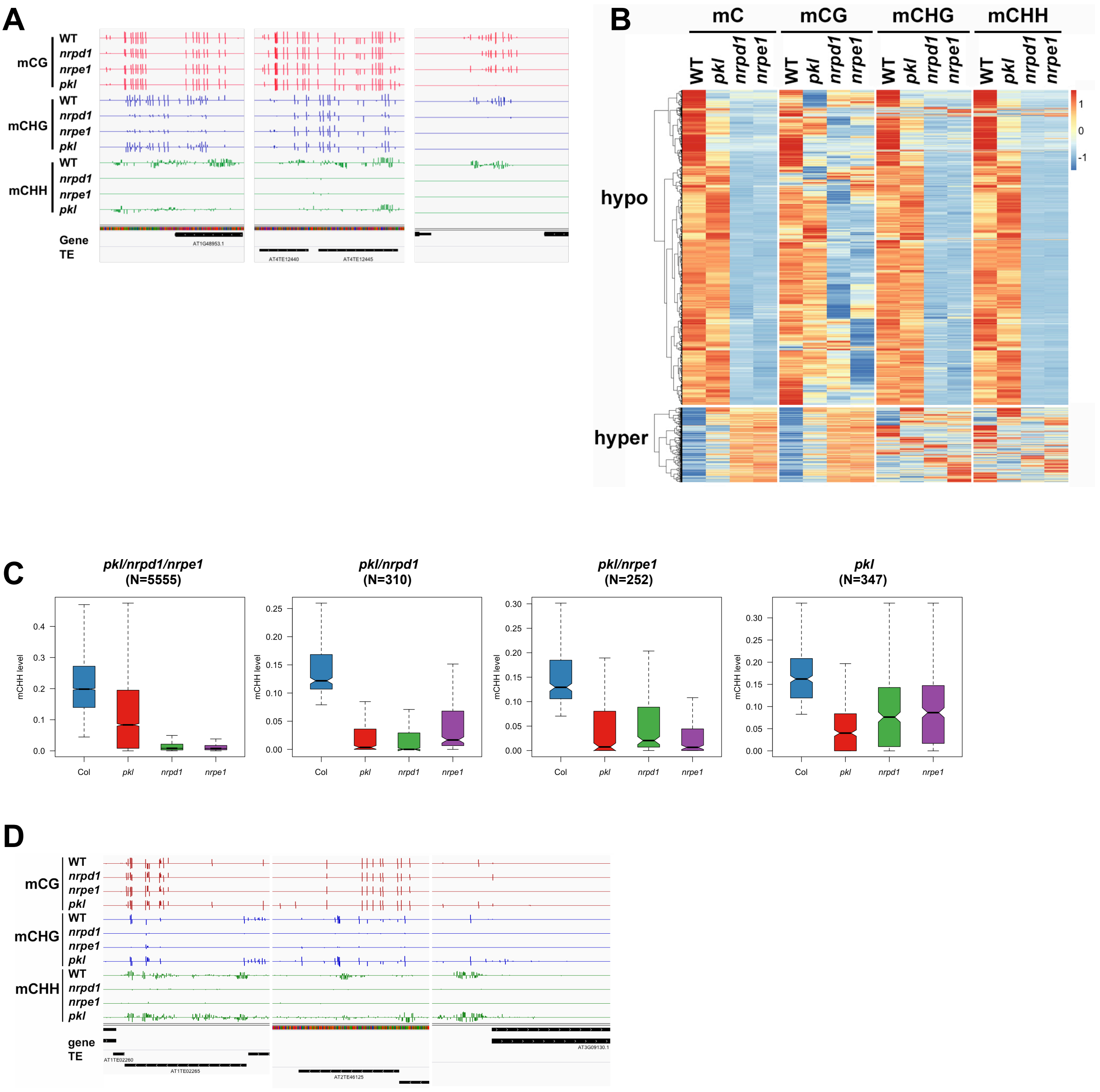

Figure S3. Characterization of differentially methylated regions in the *pkl* mutant. (A) Examples of hypoDMRs identified in the *pkl* mutant. Snapshots from the IGV (Integrative Genomics Viewer) genome browser were shown. The colored bars (red, blue, green) each represent methylation levels of cytosines in different sequence contexts (CG, CHG and CHH). All the DNA methylation levels are presented on a scale from -1 to +1, with minus values indicating cytosine methylation ratios on the minus strand. (B) Heatmap showing the relative DNA methylation levels at RdDM loci, defined by DMRs identified in the *nrpd1* and *nrpe1* mutant. The DNA methylation ratio of each DMR in the 4 genotypes was scaled to mean 0 and standard deviation 1 and ploted. (C) Distribution of CHH methylation levels at the 4 parts of CHH hypoDMRs identified in *pkl* (Figure 3C). (D) Examples of hyperDMRs identified in the *pkl* mutant. Snapshots from the IGV (Integrative Genomics Viewer) genome browser were shown.

Figure S4

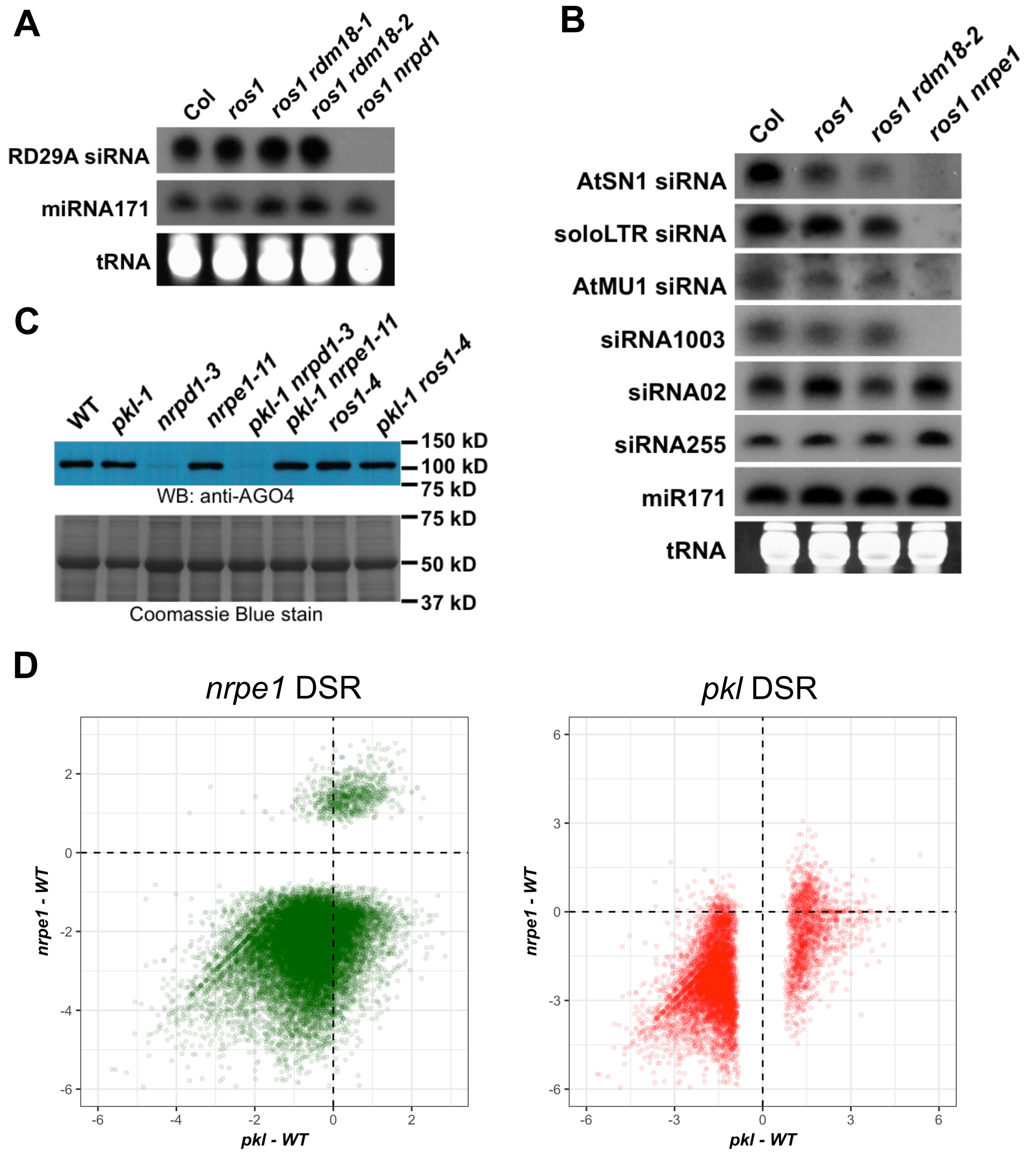

Figure S4. Effects of *pkl* on 24-nt siRNA levels and AGO4 protein levels. (A) Small RNA northern blot was used to examined the level of 24-nt siRNAs generated from the *RD29A* promoter. (B) Examination of 24-nt siRNA levels at typical RdDM loci using northern blot. The ethium bromide stained total RNA (tRNA) and miR171 serve as loading controls. (C) Anti-AGO4 western blot using total protein extracts from plants with indicated genotypes. The commassie blue-stained gel serves as a control for equal loading. Size of the protein markers were indicated on the right. (D) Scatter plots showing the relationship between the 24-nt siRNA level changes in *pkl* (x-axis) and in *nrpe1* (y-axis). Log transformed RPTM values at the *nrpe1* DSRs or *pkl* DSRs were plotted.

Figure S5

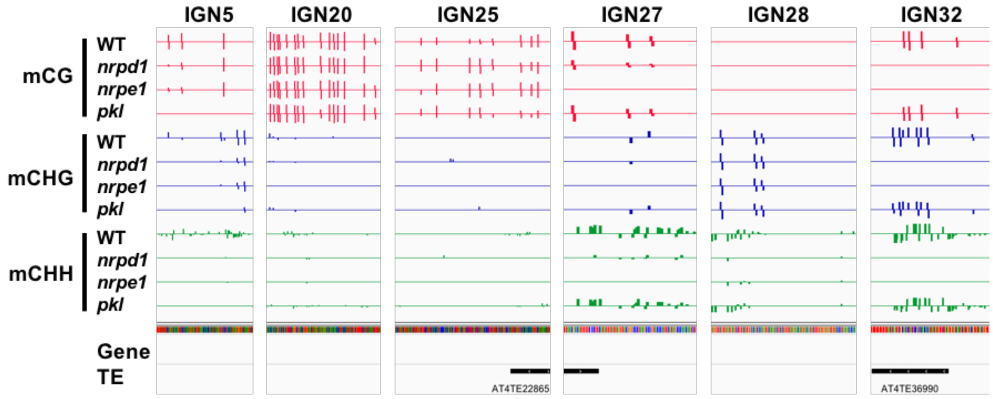

Figure S5. Screen shots of IGV (Integrative Genomics Viewer) showing DNA methylation levels at the same 6 IGN loci as in Figure 5A. The colored bars (red, blue, green) represent the methylation levels of specific cytosines on the DNA double strands.

Figure S6

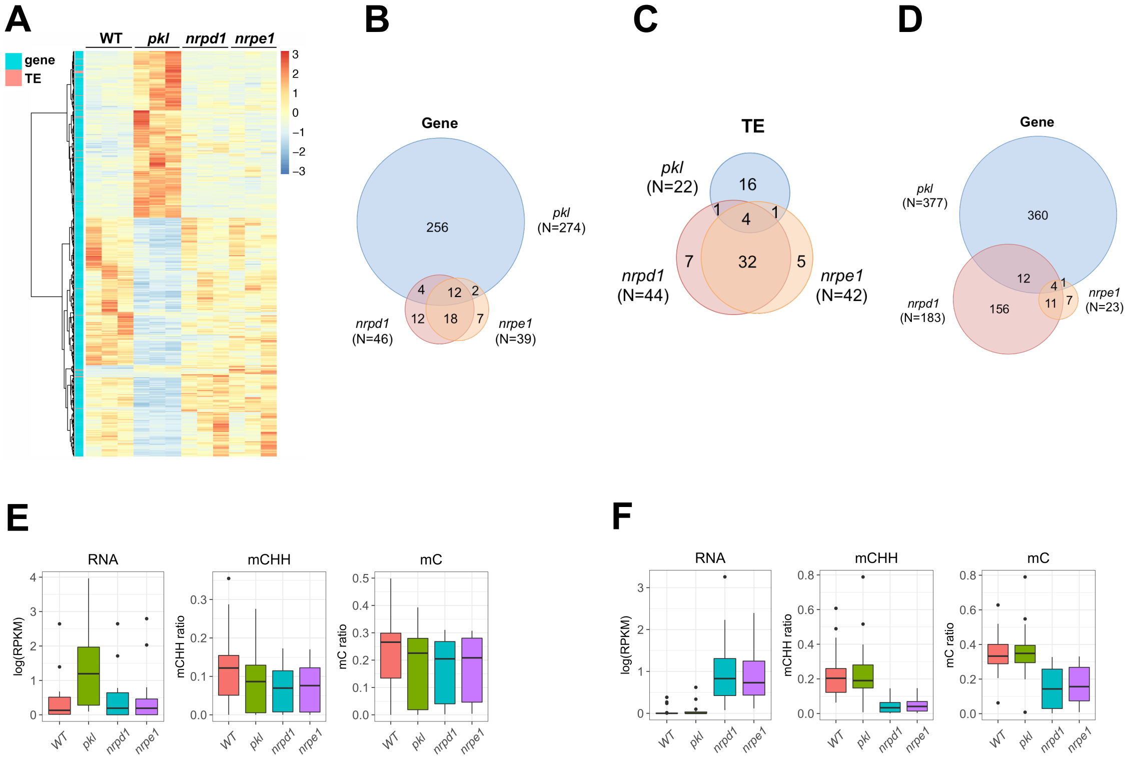

Figure S6. Analyses on TEs and genes that are differentially expressed in mutants. (A) Heatmap showing relative transcript levels of genes and TEs that were identified as differentially expressed in the *pkl* mutant. (B) Overlaps among up-regulated genes that were identified in *pkl*, *nrpd1* and *nrpe1*. (C) Overlaps among de-repressed TEs that were identified in *pkl*, *nrpd1* and *nrpe1*. (D) Overlaps among genes that are down-regulated in *pkl*, *nrpd1* and *nrpe1*. (E) Boxplots of the mRNA and DNA methylation levels of the 16 TEs that are derepressed in *pkl* but not in RdDM mutants. (F) Boxplots of the mRNA and DNA methylation levels of the 32 TEs that are derepressed in both *nrpd1* and *nrpe1* but not in *pkl*.

Figure S7

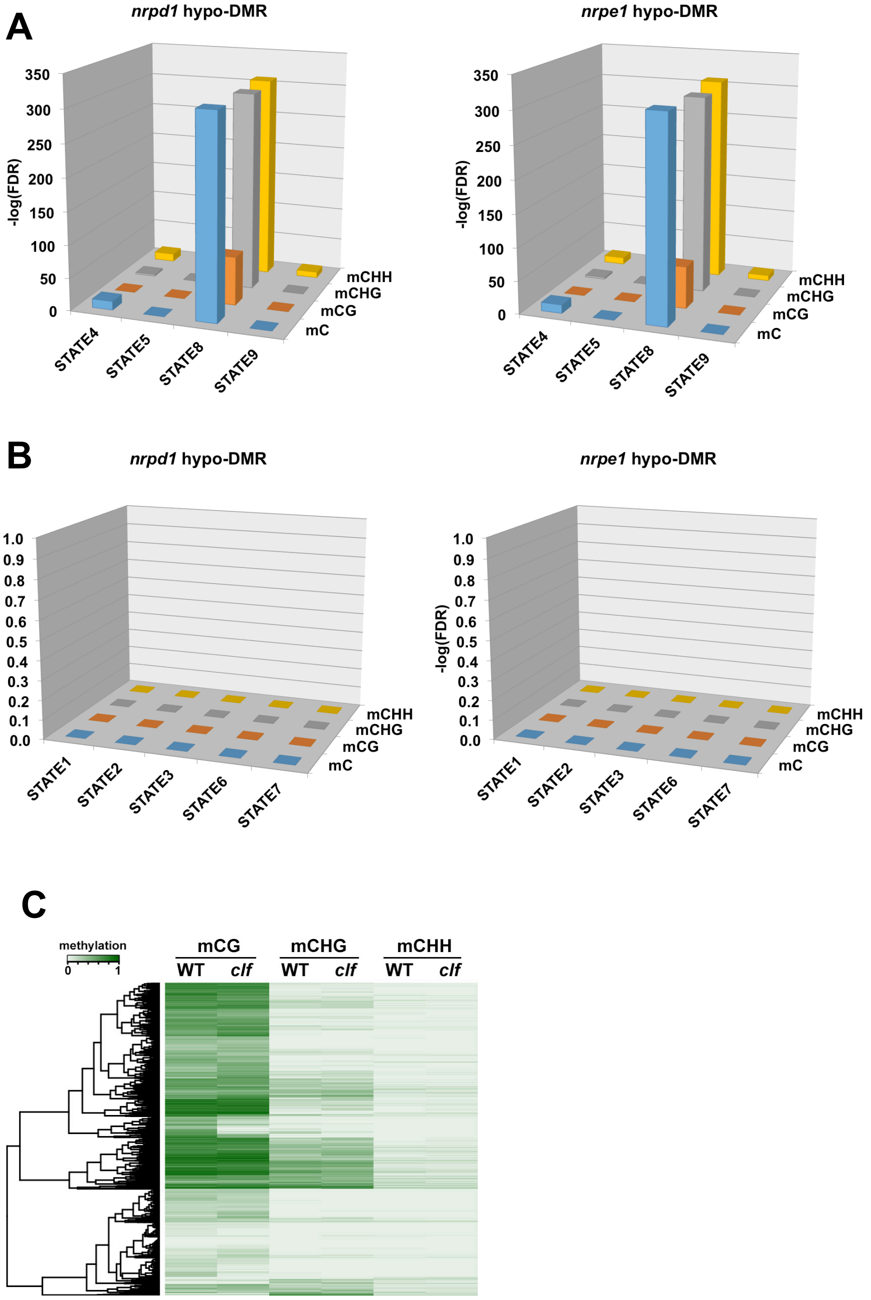

Figure S7. Chromatin features of the RdDM loci. (**A** and **B**) The log transformed FDR values (-log10) for the interaction between RdDM loci (hypo-DMRs identified in *nrpd1-3* or *nrpe1-11*) and (A) the repressive chromatin states (state 4, 5, 8, 9) and (B) the transcriptionally active chromatin states (state 1, 2, 3, 6, 7). (**C**) DNA methylation levels of the wild type and *clf* -29 plants at the genomic regions where methylation decreased in *pkl* (hypoDMRs).
